# Supplementary figures and images for: Experiment on compaction of air-dried soil under drop shocks
Source: PLoS One. 2021 Apr 15;16(4):e0250076. doi: 10.1371/journal.pone.0250076 (PMC8049244; doi:10.1371/journal.pone.0250076)

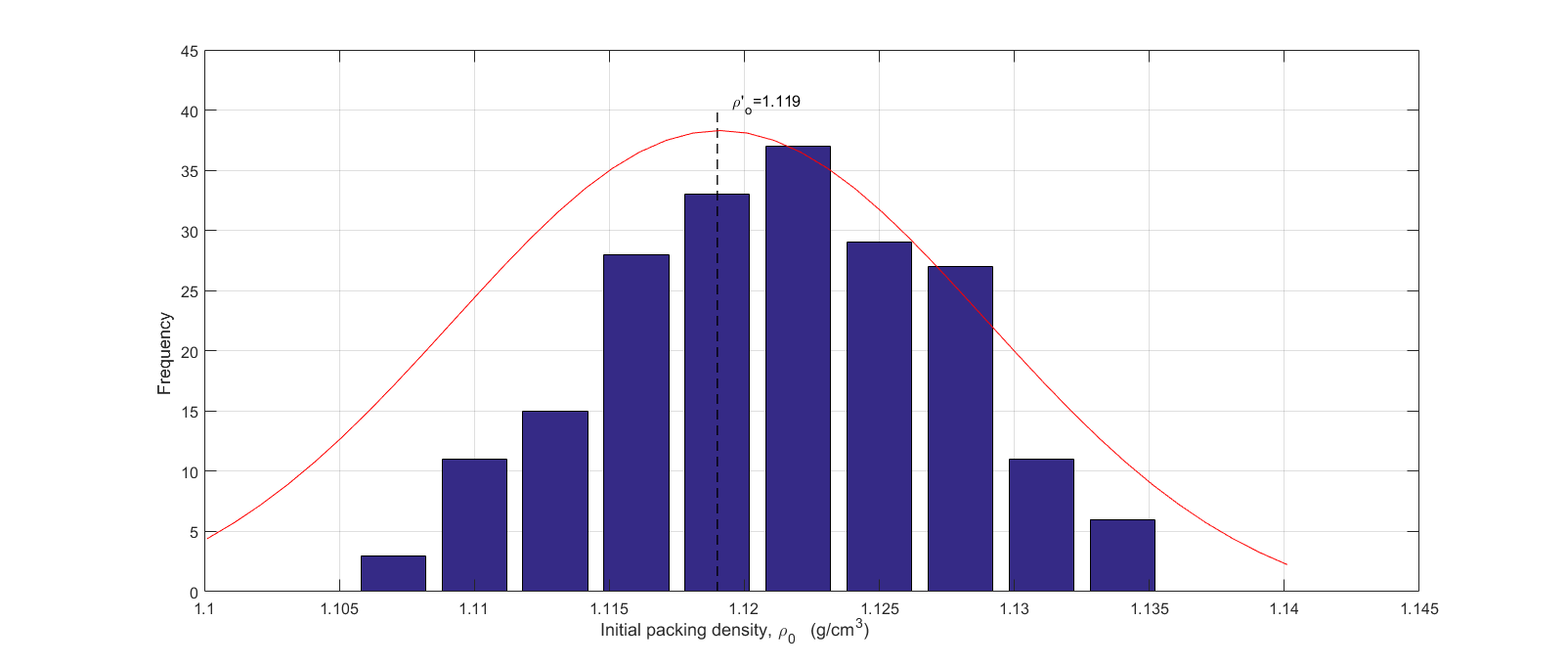

Supplement: S1 Fig — (TIF) [file pone.0250076.s001.tif]
